# Increased Iron Stores Correlate with Worse Disease Outcomes in a Mouse Model of Schistosomiasis Infection

**DOI:** 10.1371/journal.pone.0009594

**Published:** 2010-03-09

**Authors:** Cameron J. McDonald, Malcolm K. Jones, Daniel F. Wallace, Lesa Summerville, Sujeevi Nawaratna, V. Nathan Subramaniam

**Affiliations:** 1 Membrane Transport Laboratory, Division of Cancer and Cell Biology, Queensland Institute of Medical Research, Brisbane, Queensland, Australia; 2 Parasite Cell Biology Laboratory, Division of Infectious Diseases and Immunology, Queensland Institute of Medical Research, Brisbane, Queensland, Australia; 3 School of Veterinary Science, The University of Queensland, Brisbane, Queensland, Australia; 4 Liver Research Centre, School of Medicine, The University of Queensland, Brisbane, Queensland, Australia; Louisiana State University, United States of America

## Abstract

Schistosomiasis is a significant parasitic infection creating disease burden throughout many of the world's developing nations. Iron deficiency anemia is also a significant health burden resulting from both nutritional deficit as well as parasitic infection in these countries. In this study we investigated the relationships between the disease outcomes of *Schistosoma japonicum* infection and iron homeostasis. We aimed to determine if host iron status has an effect on schistosome maturation or egg production, and to investigate the response of iron regulatory genes to chronic schistosomiasis infection. Wild-type C57BL/6 and *Transferrin Receptor 2* null mice were infected with *S. japonicum*, and sacrificed at the onset of chronic disease. *Transferrin Receptor 2* null mice are a model of type 3 hereditary hemochromatosis and develop significant iron overload providing increased iron stores at the onset of infection. The infectivity of schistosomes and egg production was assessed along with the subsequent development of granulomas and fibrosis. The response of the iron regulatory gene *Hepcidin* to infection and the changes in iron status were assessed by real-time PCR and Western blotting. Our results show that *Hepcidin* levels responded to the changing iron status of the animals, but were not significantly influenced by the inflammatory response. We also show that with increased iron availability at the time of infection there was greater development of fibrosis around granulomas. In conclusion, our studies indicate that chronic inflammation may not be the primary cause of the anemia seen in schistosomiasis, and suggest that increased availability of iron, such as through iron supplementation, may actually lead to increased disease severity.

## Introduction

Schistosomiasis is a significant parasitic infection creating considerable disease burden in many tropical and developing nations. Currently, some 74 countries are endemic for schistosomiasis with an estimated 600 million people residing within regions in which the parasite is prevalent [for review see 1]. Globally, approximately 207 million people are infected with schistosomes and 120 million of these suffer chronic symptoms [2]. Iron deficiency anemia is a significant health burden resulting from both nutritional deficit as well as parasitic infection in many of these schistosomiasis-endemic nations. Chronic schistosomiasis has been associated with the burden of anemia in these developing regions for many years, although it has proven difficult to isolate and substantiate the association owing to the number of confounding factors that exist within these regions [3], [4].

Schistosomiasis in humans is caused primarily by 3 species, *Schistosoma mansoni*, *S. japonicum* and *S. haematobium*, which have different geographic distributions throughout Africa, South America and Asia. Despite differences in disease arising from the site of patent infection [5], and subtle differences in immunological responsiveness [6], [7], the host disease progression of schistosomiasis is essentially similar for all species. This similarity occurs because disease pathology does not result from infection *per se*, but rather from the intense granulomatous response to antigens secreted by the larvae within the schistosome eggs that have become lodged within host tissue [6], [7]. As the granulomatous response advances, activated hepatic stellate cells and alternatively activated macrophages are believed to be responsible for the deposition of extracellular matrix resulting in hepatic fibrosis surrounding the granulomas [8]–[10]. This fibrosis can lead to chronic obstruction of the vasculature, and in humans result in periportal fibrosis, which is responsible for severe long term disease outcomes [5]. Despite having developed a significant understanding of the pathological development of the schistosomiasis disease state, and its association with anemia being reported anecdotally for decades, it is only in recent years that the association of schistosomiasis and anemia has been definitively linked.

Several recent cross-sectional multi-variant population cohort studies have confirmed the association of anemia and the disease state of schistosomiasis. These studies have shown that when confounding factors such as nutritional deficiencies, co-infection of other parasites, age, sex, and weight are accounted for, schistosomiasis infection correlates inversely with hemoglobin levels, and can be significantly linked to the associated anemia [for a review of the association of schistosomiasis and anemia see 3], [4]. The strongest causal relationship between schistosomiasis and anemia has been established for *S. japonicum* from the Philippines, for which several studies have demonstrated an inverse relationship between infection intensity and hemoglobin [11]–[14]. Along with the confirmed linkage of anemia to schistosomiasis have come a number of theories about its cause, though little direct evidence exists to support the anecdotal explanations which exist within the literature. Most commonly, three potential causes are offered including extra-corporeal blood loss, splenic sequestration of iron, and anemia of inflammation (AI) [3], [4].

AI has become the most commonly theorized cause of the anemia following a number of studies that have shown that increased levels of pro-inflammatory cytokines, in particular IL-6, are produced during chronic schistosomiasis [12]–[16]. At significant levels, the pro-inflammatory cytokines which are produced as part of the inflammatory response, such as IL-6 and TNF-α, can lead to AI [17], [18]. It is of note though, that the association between pro-inflammatory cytokines and infection only occurs at the most intense levels of infection and hepatic fibrosis [12]–[16]. In spite of the fact that AI is often put forward as either the cause or as the major contributing factor toward the anemia, no research has investigated the response of the iron homeostatic and regulatory pathways to patent or chronic schistosomiasis. Thus it remains uncertain whether the level of pro-inflammatory cytokines associated with infection are sufficient to ‘override’ the influence of the homeostatic drive on the regulation of the iron homeostasis genes.

The central regulator of iron homeostasis is hepcidin, a 25 amino acid peptide expressed and secreted by hepatocytes. Hepcidin is up-regulated in mammals by iron overload and is suppressed by iron deficiency [for review see 19]. In iron deficient states, hepcidin transcription is down-regulated resulting in increased iron absorption by duodenal enterocytes and the release of iron stores from hepatocytes [19]. The transcription of hepcidin can also be influenced by inflammation induced by microbial pathogens and chemical stimulants, a response which forms part of an innate strategy of the host to combat infection by reducing the bioavailability of iron to the pathogens. In this instance, hepcidin is up-regulated and results in reduced iron absorption by the gut and sequestration of iron into storage in the liver and macrophages [17], [18], [20]. This has been shown in malaria patients in whom slight increases in hepcidin levels lead to marked impairment of iron incorporation into hemoglobin [21]. Unlike humans who express only a single hepcidin gene, mice express two isoforms of hepcidin, *Hepcidin 1 (Hamp1) and Hepcidin 2 (Hamp2)*. While the individual role of hepcidin 2 has not yet been confirmed, hepcidin 1 has been shown to function as the primary iron regulatory molecule. Since anemia in schistosomiasis is attributed primarily to AI, and a key mediator of AI is hepcidin [20], we undertook a detailed study of hepcidin and iron regulation in experimental schistosomiasis to determine whether anemia could be attributable solely to AI and to examine the effect of schistosomiasis on the role of iron regulatory molecules.

Hereditary hemochromatosis is a genetically heterogeneous group of iron overload disorders caused by mutations in molecules involved in the regulation of iron homeostasis. Mutations in the gene encoding hepcidin (*HAMP*) or upstream regulators of hepcidin such as hemojuvelin (*HJV*), *HFE* and transferrin receptor 2 (*TFR2*) lead to low levels of circulating hepcidin in relation to iron stores and consequent iron loading [for review see 22]. Knockout and transgenic mice have been developed that confirm the importance of these molecules in iron homeostasis and hepcidin regulation. These knockout mice represent animal models of dysfunctional iron regulation. We have previously generated *Transferrin Receptor 2* null mice (*Tfr2^−/−^*), a model of type 3 hereditary hemochromatosis and shown that they have dysregulation of hepcidin resulting in iron overload [23]. Detailed analysis of the effects of *Tfr2* dysfunction on iron homeostasis demonstrate increased iron absorption within the gut along with increased expression of iron transport genes [24].

In this study we investigated the relationship between the disease outcomes of *S. japonicum* infection, iron status and regulation. We aimed to determine if the host iron status has an effect on schistosome maturation or egg production, and to investigate the response of the iron regulatory gene *Hepcidin* to chronic schistosomiasis infection. Wild-type C57BL/6 and *Tfr2^−/−^* mice were infected with *S. japonicum*, and sacrificed at the onset of chronic disease. The infectivity of schistosomes and egg production was assessed along with the subsequent development of granulomas and fibrosis. The response of the iron regulatory gene *Hepcidin* to infection and the changes in iron status was assessed within the mice. We show that *Hepcidin* responds to the changing iron status of the animals but is not significantly influenced by the inflammatory response. We also show that with increased iron levels in the host pre-infection there was greater development of fibrosis around granulomas. Our studies indicate that chronic inflammation may not be the primary cause of the anemia seen in schistosomiasis. Further we demonstrate that increased iron at the onset of infection leads to increased liver fibrosis resulting in a more severe long term outcome.

## Results

### Iron Status with Infection

**Figure 1:**
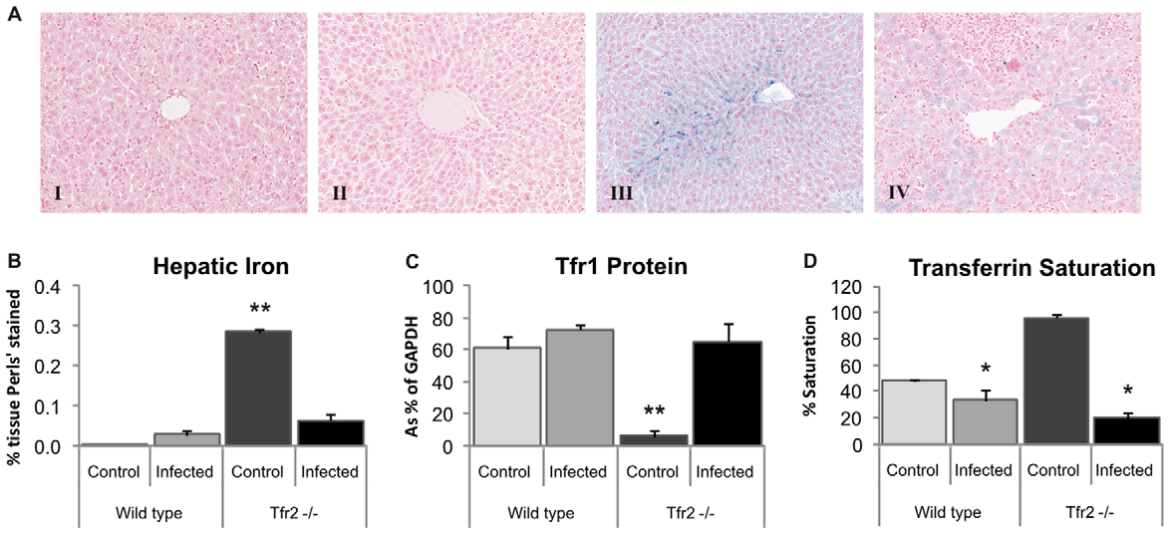
Schistosomiasis infection results in a significant reduction in iron status. Perls' staining as a measure of hepatic iron, Tfr1 protein levels, and serum transferrin saturation were measured in wild-type and *Tfr2^−/−^* control and schistosome infected mice. A. Perls' Prussian Blue staining of liver sections shows the various levels of hepatic iron stores; (I) wild-type control, (II) wild-type infected, (III) *Tfr2^−/−^* control, (IV) *Tfr2^−/−^* infected. B. Quantification of Perls' Prussian Blue staining for non-heme iron within hepatocytes showed that while significantly more iron was present in *Tfr2^−/−^* uninfected animals, the level was reduced to levels similar to wild-type mice in infected *Tfr2^−/−^* animals. C. Quantification of Western blotting for Tfr1 protein from hepatocytes shows the expected inverse down-regulation of the protein in the *Tfr2^−/−^* uninfected animals with high iron stores. D. Serum transferrin saturation is significantly lower in both wild-type and *Tfr2^−/−^* infected mice when compared to uninfected controls. (*P<0.001 compared to respective control group; **P<0.001 compared to all others; Error bars ± SEM).

At the time of parasite perfusion, hepatic iron stores were estimated by quantification of Perls' Prussian Blue staining for non-heme iron within liver sections (figure 1A and B). The quantification of staining shows that only uninfected *Tfr2^−/^* ^−^ control animals had hepatic iron levels significantly above background with 0.28% of tissue stained with Prussian Blue (P<0.001). We next assessed the level of transferrin receptor 1 (*Tfr1*) protein in livers. Tfr1 is responsible for the uptake of transferrin-bound iron into hepatocytes and its expression level is inversely regulated by the cellular iron content. Tfr1 protein was significantly lower in uninfected *Tfr2^−/−^* control mice (figure 1C; P<0.001). Given the inverse relationship between Tfr1 protein and hepatocyte iron content, this Tfr1 profile reflects the iron content estimated by quantification of the Perls' Prussian Blue staining (Figure 1B).

Uninfected *Tfr2^−/−^* control mice show significantly (P<0.001) higher serum transferrin saturation (TS), as representative of circulating iron, than wild-type animals with 96% and 48% saturation respectively (figure 1D). In *S. japonicum* infected mice TS was significantly (P<0.05) lower in both *Tfr2^−/−^* and wild-type animals when compared to their respective uninfected controls groups, a reduction of 76% and 14% respectively. Together these results show that infection significantly reduces the iron status of both strains of mice. The iron over-loaded status of the *Tfr2^−/−^* mice, as shown by the Perls' quantification, Tfr1 protein levels and TS provided the background to compare the effects of iron availability on schistosome maturation, egg production, and liver pathology.

### Effects of Iron Status on *Schistosoma* Maturation and Egg Production

**Figure 2:**
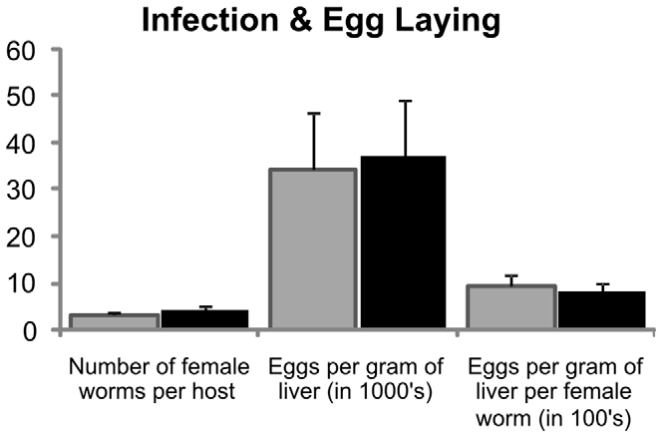
Iron status has no effect on *S. japonicum* maturation or egg production. Wild-type and *Tfr2^−/−^* mice were sacrificed 6 weeks after infection with *S. japonicum.* The number of worm pairs perfused from the portal vein was then counted and the number of eggs per gram of liver was calculated following egg recovery from liver samples. In spite of significantly higher levels of iron stores in the *Tfr2^−/−^* mice, this did not affect the number of worms which matured or the number of eggs produced either in total or per female (Light grey wild-type, black *Tfr2^−/−^*; Error bars ± SEM).

In order for schistosome cercariae to mature into adult worms capable of egg production they require substantial iron which they must sequester from their host [25]. In spite of there being significantly more iron available at the time of infection to schistosomes in *Tfr2^−/−^* mice than in wild-type mice, there was no statistically significant difference in the number of mature females (figure 2). Adult female worms use significant amounts of iron for egg-shell production [26], and it could be expected that iron availability may be a limiting factor in egg production. However, there was no significant difference observed in the number of eggs per gram of liver, or the number of eggs per gram of liver per female worm (figure 2).

### Interaction between Iron Status, Inflammatory Response, and Iron Regulatory Genes

**Figure 3:**
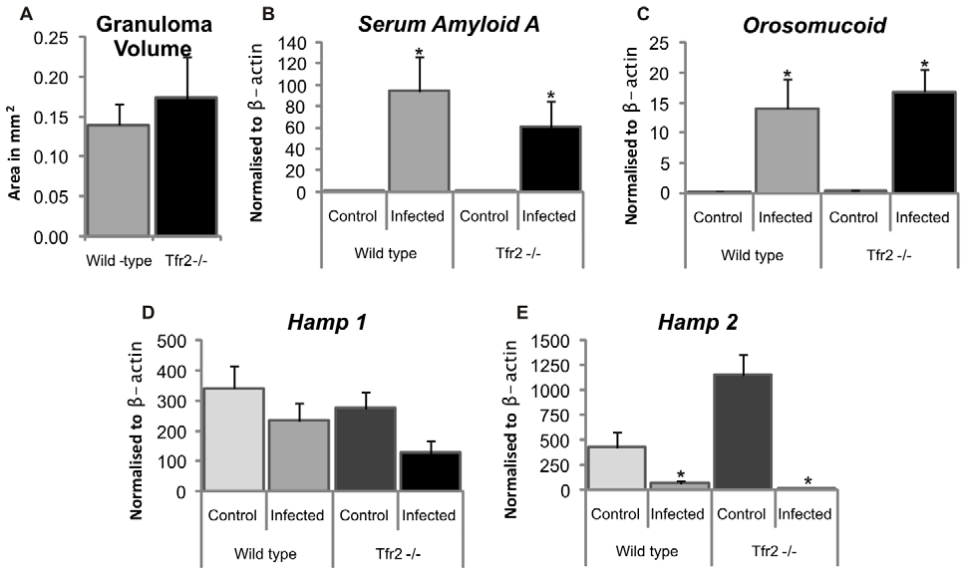
Interaction of iron status, inflammation, and iron regulatory genes. The response of inflammatory markers and iron regulatory genes was assessed in schistosomiasis infected and control wild-type and *Tfr2^−/−^* mice 6 weeks post infection date. Granuloma size was estimated by point-counting stereology on liver sections, and gene expression was assessed by quantitative real-time PCR from liver RNA. A. Comparison of the average granuloma area within wild-type and *Tfr2^−/−^* mice shows no difference in response in infected animals. B and C. Hepatic inflammatory markers *Serum Amyloid-A* and *Orosomucoid* show a significant response to infection, but no difference in the levels of response between wild-type and *Tfr2^−/−^* animals reflecting granuloma size. D and E. *Hamp 1* shows a trend and *Hamp 2* shows significant down-regulation in response infection. This pattern of regulation is consistent with the identified iron status of the animals and appears unaffected by the inflammatory response identified (*P<0.05 compared to respective control group; Error bars ± SEM).

The liver pathology of infected hosts develops as a result of the immune inflammatory response to egg antigens, a response which leads to granuloma formation around the lodged eggs resulting from progressive immune cell infiltration and accumulation. Thus measurement of granuloma size can provide a measure of the intensity of the host's inflammatory immune response to egg antigen. In this aspect of host response, there was no difference between the average sizes of the granulomas produced in wild-type and *Tfr2^−/−^* mice (figure 3A).

*Serum Amyloid-A* (*SAA*) and *Orosomucoid* (*Orm*; also known as *α-1 acid glycoprotein (AGP*)) are acute phase response genes expressed by hepatocytes and are representative of hepatic inflammation [27]. These markers show that significant hepatic inflammation occurred in both wild-type infected and *Tfr2^−/−^* infected mice, but not in uninfected control mice (figure 3B and C). There was no significant difference in the expression levels of these hepatic inflammatory markers between wild-type and *Tfr2^−/−^* for either infected or uninfected mice, consistent with the similarity in granuloma size between the two groups.

Expression levels of *Hepcidin 1 (Hamp1)* displayed a downward trend in infected mice of both strains, while *Hepcidin 2 (Hamp2*) expression was significantly reduced in both strains with infection (figure 3D and E). The *Hamp1* levels in *Tfr2^−/−^* control mice are lower than that of wild-type controls in spite of having significantly higher iron levels as a consequence of *Tfr2* knockout and is the expected phenotype for these animals [23]. Down-regulation of *Hamp1*, as seen in infected animals, results in increased absorption of iron in the gut and liberation of stored iron, thus representing a normal response to the reduced iron status of the infected animals. As the specific role of *Hamp2* has not yet been clarified, it cannot be determined if its down regulation is representative of a response to the iron status of the animal or a response to the infection itself.

### Effects of Iron Status on Hepatic Fibrosis

**Figure 4:**
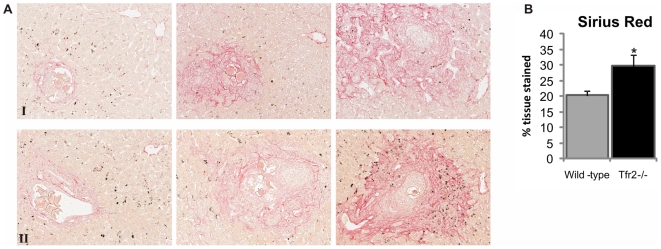
Iron status affects development of fibrosis. The development of fibrosis surrounding granulomas was measured in the livers of schistosomiasis infected and uninfected wild-type and *Tfr2^−/−^* mice 6 weeks post infection date by quantification of the Sirius Red staining of collagen. A. Sirius Red staining shows the varying dispersion of the collagen within individual wild-type (panel I) and *Tfr2^−/−^* animals (panel II). Brown dots represent lipofuscin accumulated in autophagolysosomes. B. Quantification of Sirius Red staining of collagen indicating development of fibrosis shows greater deposition within *Tfr2^−/−^* animals than in wild-type animals. (*P<0.05; Error bars ± SEM).

Progression of the granulomatous response leads to the deposition of collagen in the tissue surrounding the granuloma. The degree of fibrosis development is highly dependent on the length of time the eggs have been lodged in the tissue as the extent of collagen deposition varies greatly between granulomas even within individual animals (figure 4A). In spite of this variability, and even at the early stage of chronic disease onset in infected animals, significantly (P<0.05) more collagen was detectable in the livers of infected *Tfr2^−/−^* mice than infected wild-type mice with 29.7% and 20.2% of tissue stained with Sirius Red respectively (figure 4B). Brown dots seen within the tissue section represent lipofuscin accumulation in autophagolysosomes.

## Discussion

In this study we show that schistosomiasis infection resulted in a substantial reduction in iron indices, with infected wild-type and *Tfr2^−/−^* mice having significantly lower TS and hepatic iron than uninfected mice. This reduction in iron status reflects the phenotype seen in human patients, a disease outcome most commonly attributed to AI. In general, AI results from two processes induced in response to inflammation: first, iron may be sequestered within tissues reducing its availability within the body, and second, iron absorption by gut enterocytes is blocked thereby eliminating the dietary source of iron [reviewed in 28]. In the model used in this study (10 week old C57BL/6 mice) infected mice were studied 6 weeks post-infection, approximately 1–2 weeks after the onset of parasite egg laying, their entrapment in the liver and the induction of host granulomatous response. The intense inflammatory response directed against eggs might be expected to lead to AI, typified by an inhibition of iron absorption through the up-regulation of hepcidin [6]. *Schistosoma japonicum* infection in these mice, however, led to down-regulation of hepcidin (as measured by expression levels of *Hamp1 and 2*). Decreased hepcidin expression results in increased iron absorption, the opposite of what is required for inflammation to result in AI [20]. Thus, it is unlikely in this murine infection that decreased iron absorption induced by AI contributed significantly to the reduced iron status of the infected animals. Moreover, liver iron stores were decreased in these schistosomiasis-infected mice, whereas in other infections liver iron stores are increased in AI [28]. Thus iron sequestration by normal pathways can be excluded as a cause of AI in schistosomiasis. It is therefore unlikely that AI is the primary cause of the reduced iron status associated with the schistosomiasis infection.

Friis and co-workers investigated the use of macro-nutrient supplements in treatment of Kenyan school children suffering chronic *S. mansoni* infection [29]. Their study found macro-nutrient supplementation improved hemoglobin levels to the same level observed with treatment with the anti-schistosome drug praziquantel. Both macro-nutrient supplementation alone and praziquantel treatment alone resulted in significantly higher hemoglobin levels than for subjects given placebo, bringing hemoglobin levels of treated individuals into the normal range (13–18 mg/dL). Our studies, which show decreased hepcidin expression, with likely increase in absorption of dietary iron, provides experimental evidence in support of Friis and colleagues. In the situation of AI however the opposite occurs, hepcidin is up-regulated decreasing the ability of the gut to absorb iron from the diet. In the presence of AI, macro-nutrient supplementation should not be effective at increasing hemoglobin levels.

Within the schistosomiasis literature the presence of anemia is often attributed to AI because of the failure of occult blood loss to account for the anemia [14], and the detectable levels of inflammatory markers. Leensta *et al* suggested that AI is likely to be a significant contributor to the anemia seen in schistosomiasis following their findings that anemia was commonly present in the absence of iron deficiency (ID) [12], [13]. However, in their study the absence or presence of ID was assigned to subjects on the basis of their serum ferritin (SF) levels. A literature review conducted by WHO in 2007 reported that the use of SF levels in the presence of inflammation is likely to result in significant misclassification of ID subjects as iron replete [30]. WHO now recommends that SF is not a useful indicator of ID when inflammation is present [30]. Therefore, it is possible that in the study of Leensta *et al*, subjects with anemia who were classified as iron replete based on their SF may have actually been ID.

In another cohort study, Cherian and co-workers investigated the relationship of hepcidin and iron status in refugee children [31]. They demonstrated that hepcidin levels among children with ID correlated with the iron status, and not the infection or inflammatory status of the child. Furthermore, in this study ID was defined on the basis of ≥2 abnormal age-corrected iron parameters, assisting in better identification of ID in the presence of infection and inflammation [31]. These findings support our results which show that hepcidin, the master regulator of iron homeostasis and the key regulatory gene responsible for AI is not affected by the levels of infection and inflammation seen in these studies.

*Tfr2^−/−^* mice have a greater amount of bioavailable iron for invading schistosomes than wild-type mice at the time of infection. While we did not measure iron absorption and thus iron availability directly during infection in our study, previous studies on *Tfr2* dysfunction in mice have demonstrated increased iron absorption through the gut along with increased expression of iron transport genes [24]. Further to this we have demonstrated decreased *Hepcidin* expression concurrent with *S.japonicum* infection. It is therefore likely that elevated dietary iron absorption occurs concurrently with infection, providing ongoing increased iron availability within the *Tfr2^−/−^* host animals. Despite the high requirements of invading schistosomules for host iron, and an expectation that the greater availability of iron in *Tfr2^−/−^* mice might lead to higher burdens in those mice, there were no differences in adult worm numbers between wild-type and *Tfr2^−/−^* mice. The progression toward iron depletion in schistosomiasis may be initiated with the onset of egg-laying, which occurs approximately 4 weeks post infection for *S. japonicum*, resulting in transfer of iron from the host into the eggs. If on-going iron availability is a rate-limiting factor in the progression toward some aspect of chronic disease, then the level of a host's pre-infection iron stores may contribute to the rate of onset of severe symptoms, i.e. the larger the available iron pool, the faster the onset of chronic symptoms. In the same manner, iron supplementation to resolve the anemia associated with schistosomiasis may actually increase the development of long term pathology by providing on-going increased iron availability.

Ironically the anemia associated with schistosomiasis may actually provide a degree of protection for hosts against the more severe long term pathological consequences of chronic infection. The development of fibrosis surrounding granuloma formation can lead to significant health consequences resulting in morbidity and mortality. We showed that infected *Tfr2^−/−^* mice with high iron indices develop a significantly increased fibrotic response surrounding the granulomas when compared to infected wild-type mice. Activated hepatic stellate cells (HSC) and alternatively activated macrophages are responsible for the deposition of extracellular matrix resulting in hepatic fibrosis surrounding the granulomas [8]–[10]. Ramm *et al* have also shown a direct correlation between the hepatic iron concentration (HIC) and the activation of HSCs [32], showing that increased HIC increases both the activation of HSCs prior to the deposition of collagen, and the amount of collagen deposited [32]. This correlation between HIC and fibrosis is believed to result from increasing exposure to the oxidative stress resulting from intracellular iron [32]. As such, the reduced HIC status of schistosomiasis infected mice may result in a decreased level of HSC activation, which in turn results in lower deposition of collagen.

Denic and Agarwal propose that iron deficiency provides an evolutionarily-selected survival advantage [33]. They suggest that following the agricultural revolution which resulted in significantly decreased dietary iron intake, iron deficiency provided a survival advantage to those with endemic diseases such malaria, plague and tuberculosis [33]. Further to this, other studies such as the correlation of HIC and fibrosis, suggest that iron deficiency may also provide a survival advantage in chronic disease states including schistosomiasis.

The disease outcomes associated with schistosomiasis have a significant impact throughout many of the world's developing nations. The anemia associated with schistosomiasis is often labeled as anemia of chronic inflammation: however our findings suggest that this is likely not to be the case. We show that the iron regulatory gene *Hepcidin* responds to the decreased iron status. Furthermore, we have shown than increased iron available from pre-infection stores and possible ongoing iron absorption leads to increased fibrotic deposition within the liver. Together these results suggest that increasing the iron status of infected subjects may actually result in an increased disease burden. In light of this the utilization of dietary iron supplementation to resolve anemia associated with chronic schistosomiasis may result in more severe long term disease outcomes.

## Materials and Methods

### Animals

All mice (female; 10 weeks of age) were maintained on standard laboratory chow (Norco Stockfeeds, South Lismore, Australia). Wild-type C57BL/6 mice were purchased from the Animal Resource Centre, Perth, Australia. *Tfr2^−/−^* mice on the C57BL/6 background were bred in-house and have been described previously [23] All animals received humane care according to the criteria outlined in the “Guide for the Care and Use of Laboratory Animals” prepared by the National Institutes of Health [34]. All experimental protocols were approved by the Queensland Institute of Medical Research Animal Ethics Committee.

### Animal Infection

*Oncomelania hupensis hupensis* snails, infected with Chinese (Anhui) strain of *S. japonicum*, were imported from the Institute of Parasitic Diseases, Shanghai, China. Mice were infected percutaneously with 30 cercariae shed from snails, and adult worms were perfused 6 weeks after challenge as described previously [8]. Livers were removed from mice for egg counts and histology as described [8].

### Histochemistry and Measurement of Iron Indices

Liver tissue was fixed in 4% formalin for 24 hours prior to embedding in paraffin wax and sectioning. Fibrosis was detected in sections by staining for collagen with Sirius red which was performed by the histology department at QIMR. Iron was detected in sections using the Perls' Prussian Blue staining method. Briefly, liver sections were deparaffinized and rehydrated. Slides were incubated in Perls' Staining Solution (comprising equal parts of potassium ferrocyanide and HCl), washed, counterstained with Nuclearfast Red, dehydrated, cleared in xylene and mounted using Depex mounting medium. Transferrin saturation (TS) was measured using an iron and iron binding capacity kit (Sigma-Aldrich, Castle Hill, Australia). Sections were scanned using the Aperio ScanScope XT (Aperio, Brisbane, Australia) with doubler inserted for x40 scanning. Sirius Red and Perls' Prussian Blue stains were quantified within the sections using the ImageScope Version 10.0.36.1805 software and the ColorDeconvolution_v9 algorithm calibrated to the sections.

### Real-Time PCR

Total RNA from mouse liver was isolated using Trizol (Invitrogen, Mulgrave, Australia) and treated with DNase to remove any genomic DNA. One µg was reverse transcribed into cDNA with Superscript III (Invitrogen, Mulgrave, Australia). Real-time reaction mixes contained cDNA transcribed from 15 ng RNA, 200 nM each primer and LightCycler 480 SYBR Green Mix (Roche, Brisbane, Australia). Reactions were performed on the LightCycler 480 (Roche, Brisbane, Australia) using the following conditions: 2 minutes denature at 95°C followed by 45 cycles of 95°C for 15 seconds, 55°C for 15 seconds and 72°C degrees for 15 seconds. The fluorescence due to SYBR green binding to double stranded DNA was measured during the extension steps. Melt curve analysis was routinely performed to monitor primer dimer levels by raising the temperature from 50°C to 99°C at 1 degree/minute whilst monitoring fluorescence continuously. All experiments were performed in duplicate, and a single batch of cDNA was used for *β-actin*, *Hamp1*, *Hamp2*, *Tfr1*, *Tfr2* [23], *Orm* (forward: TGGAAGCTCAGAACCCAGAA, reverse: AGCCGCACCAATGAAAAAC), *Saa* (forward: AGTGGCAAAGACCCCAATTAC, reverse: GGTAGGAAGAAGCCCAGACC). All targets were normalized to the respective β-actin levels by subtracting the threshold cycle (CT) of *β-Actin* from the CT of the target (ΔCT). Results were then transformed to log base 2 to represent fold change and normalized to 1 for comparison.

### Western Blotting

Tissues were homogenized in phosphatase inhibitor lysis buffer (200 mM Tris pH 8.0, 100 mM NaCl, 1 mM EDTA, 0.5% NP-40, 10% glycerol, 1 mM NaF, 1 mM sodium orthovandate, 1 mM sodium pyrophosphate, 1∶1000 protease inhibitor cocktail, 2 mM PMSF) containing 10 µg/mL DNase. Twenty five micrograms of total liver homogenates were electrophoresed on a 10% SDS-PAGE and then transferred onto Hybond-C+ membrane. Blots were blocked in 10% skim milk powder, 0.5% Tween 20 in PBS (blocking buffer) overnight at 4°C. One µg/mL each of anti-TfR1 (Invitrogen) or anti-actin (Sigma) antibody, diluted in blocking buffer, was applied to the blot for 1 hour at room temperature. The blots were washed extensively with 0.1% Tween 20 in PBS and then incubated with anti-rabbit or anti-mouse horse-radish peroxidase for 1 hour at room temperature. Blots were washed extensively and Immobilon Western chemiluminescent HRP substrate (Millipore WSBLKS0500) was applied for 5 minutes to the blot and then exposed to film.
